# FXYD3 Is Frequently Expressed in Pancreatic Ductal Adenocarcinoma but Does Not Predict Survival

**DOI:** 10.1002/cam4.70500

**Published:** 2025-01-09

**Authors:** Nathalie B. Rasko, Christopher B. Nahm, John Turchini, Rachel Teh, Helge Rasmussen, Sooin Byeon, Sumit Sahni, Jaswinder S. Samra, Anthony J. Gill, Anubhav Mittal

**Affiliations:** ^1^ Faculty of Medicine and Health, Sydney Medical School University of Sydney Sydney New South Wales Australia; ^2^ Upper Gastrointestinal Surgical Unit, Department of Gastrointestinal Surgery Royal North Shore Hospital Sydney New South Wales Australia; ^3^ Cancer Diagnosis and Pathology Group, Kolling Institute Royal North Shore Hospital Sydney New South Wales Australia; ^4^ Cardiac Membrane Biology Laboratory, Kolling Institute The University of Sydney Sydney New South Wales Australia; ^5^ Australian Pancreatic Centre Sydney New South Wales Australia; ^6^ Faculty of Medical and Health Sciences Macquarie University Sydney New South Wales Australia

**Keywords:** FXYD3, MAT‐8, pancreatic cancer, pancreatic ductal adenocarcinoma, prognosis, survival

## Abstract

**Background:**

FXYD3 is a Na/K‐ATPase modulator which is upregulated in pancreatic ductal adenocarcinoma (PDAC), but its prognostic role is unknown. This study evaluated FXYD3 expression in chemo‐naive patients with surgically‐resected PDAC at a single centre (1993–2014).

**Method:**

FXYD3 expression was assessed in tumour specimens using immunohistochemistry.

**Results:**

145 of 180 PDAC tumour specimens were FXYD3‐immunopositive (80.5%). There was no difference in median overall survival between the FXYD3 negative (27.60 months) and positive groups (25.00 months) (log‐rank *p* = 0.9718). FXYD3 expression correlated positively with late‐stage disease (OR 3.041, 95% CI 1.190–7.455, *p* = 0.0175). There was no significant association with T stage, positive lymph nodes, perineural invasion, lymphovascular invasion or histological grade.

**Conclusion:**

Immunohistochemical FXYD3 expression does not predict survival in chemo‐naive PDAC patients, but is associated with late‐stage disease. The high rate of FXYD3 overexpression warrants therapeutic evaluation.

## Graphical abstract

Pancreatic ductal adenocarcinoma (PDAC) remains one of the world's deadliest cancers, and there is significant imperative to develop novel prognostic biomarkers to guide our limited therapeutic options. FXYD3 is a transmembrane protein overexpressed in numerous cancers including pancreatic cancer and, like other seven members of the FXYD family, is known to modulate Na/K‐ATPase activity. However, despite knowledge of its upregulation, the role of FXYD3 in pancreatic cancer survival has not yet been assessed on a larger scale. Herein, we present a study establishing the rate of immunohistochemically‐evaluated FXYD3 expression in chemotherapy‐naïve resected PDAC tumours. Furthermore, we demonstrate that FXYD3 expression does not correlate with survival in these patients.

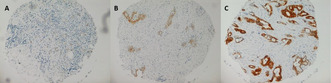

**Abbreviations**
FFPE: formalin‐fixed paraffin‐embedded
FXYD3: FXYD domain‐containing ion transport regulator
MAT‐8: mammary tumour protein 8
PDAC: pancreatic ductal adenocarcinoma
TMA: tissue microarrays

## 1 Introduction

Pancreatic ductal adenocarcinoma (PDAC) continues to pose a significant clinical challenge with a five‐year survival rate of only ~8% and marginal improvements over several decades [1, 2]. This is in part related to late diagnosis, resistance to systemic therapy and lack of therapeutic targets. Moreover, up to 35% of patients who undergo curative‐intent PDAC resection face mortality due to early recurrence of disease [3]. There is therefore a pressing need to establish and validate prognostic biomarkers for PDAC that may allow for better selection of patients for PDAC resection.

The 7‐member mammalian membrane protein FXYD family is named after the one‐letter code of an amino acid sequence they share in the extracellular domain. They are widely expressed in a tissue‐specific manner [4] and implicated in diverse functions including ion transport, cellular signalling and inflammation. In particular, FXYD proteins are known for their association with the Na/K‐ATPase and modulation of its ion transport properties [5]. The third member of this family, FXYD3, or mammary tumour protein 8 (MAT‐8), is a 8‐kDA transmembrane protein that was initially identified as an inducer of chloride conductance in *Xenopus laevis* oocytes and subsequently also shown to associate with the Na/K‐ATPase [6, 7]. FXYD3 is overexpressed in various tumours including human breast cancer [8], bladder cancer [9], prostate carcinoma [10], rectal cancer [11], gastric adenocarcinoma [12], intrahepatic cholangiocarcinoma [13] and hepatocellular carcinoma [14].

FXYD3 has also been found to be overexpressed in PDAC tumours [15, 16, 17, 18], and silencing this protein has been demonstrated to slow down the growth of PDAC cells [18]. However, the prognostic significance of immunohistochemical FXYD3 expression on patients with resected PDAC has not previously been evaluated. Given its role in potentiating PDAC growth in vitro, we hypothesised that FXYD3 expression would be associated with poorer prognosis in patients with resected PDAC.

The aims of this study were to (1) determine the rate of FXYD3 expression in PDAC tumours and (2) determine the association of FXYD3 expression with overall survival and histopathological parameters.

## 2 Materials and Methods

### 2.1 Study Design and Selection Criteria

Consecutive patients who underwent pancreatic resection at a single tertiary level institution for a histopathological diagnosis of PDAC between 1993 and 2014 were included for analysis. Patients with diagnoses of alternative periampullary malignancies (e.g., ampullary carcinoma, duodenal carcinoma, cholangiocarcinoma, acinar cell carcinoma) were excluded. Those who received neoadjuvant therapy or had operative mortality were also excluded from the analysis. The conduct of this study was performed in accordance with the World Medical Association Declaration of Helsinki. Ethical approval was obtained from the Northern Sydney Local Health District Human Research and Ethics Committee (NSLHD HREC; Master Protocol # 2019/ETH08639 and associated LNR# 2023ETH01069). A waiver of consent was obtained from NSLHD HREC to use archived tissue blocks under NSW Human Tissue Act 1983.

### 2.2 Patient Treatment and Clinical Characteristics

Pancreatic resection for the cohort consisted of one of the following operations: pancreatoduodenectomy (PD); distal pancreatectomy with or without splenectomy (DP); or total pancreatectomy (TP). Patients were routinely offered adjuvant therapy after surgery. We have previously reported the take‐up rate of adjuvant[I1] treatment at our institution as 84% [19].

Clinicopathological data including demographic information, tumour stage, tumour grade, perineural invasion and lymphovascular invasion were retrieved from a prospectively maintained database by the research team. Tumour pathological stage was determined using the AJCC 8th Edition guidelines. Survival status was determined by accessing the patient electronic medical records. The survival period was defined as the time elapsed between from the date of surgery to the date of death.

### 2.3 Immunohistochemistry

Tissue microarrays (TMAs) were formed from formalin‐fixed paraffin‐embedded (FFPE) blocks of all tumour specimens. One‐millimetre cores were taken of each tumour in duplicate using the Chemicon ATA‐100 microarrayer and re‐embedded in paraffin. The TMAs were sectioned at 4 μm onto positively charged slides (Superfrost plus, Menzel‐Glaser, Germany). Slides were then de‐paraffinised in xylene and rehydrated in graded ethanol solutions. Heat induced epitope retrieval was undertaken at pH 9.0 in a 99°C water bath for 20 min. Slides were incubated at 4°C overnight with FXYD3 primary antibody (AbCam, anti‐rabbit mAb, clone EPR17160, ab205534, 1:8000 dilution) and subsequently quenched with 0.3% hydrogen peroxide solution. Slides were then incubated at room temperature for 30 min with a secondary antibody (Dako EnVision+ Anti‐Rabbit System‐HRP labelled polymer #K4003) and then with DAB chromogen for 10 min. Slides were counterstained with Mayer's haematoxylin, dehydrated in ethanol and then mounted for scoring.

**FIGURE 1:**
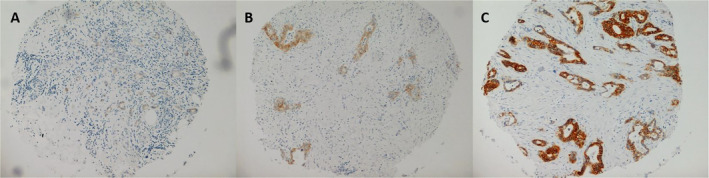
Representative examples of scored FXYD3 immunoreactivity in resected pancreatic ductal adenocarcinoma tissue microarray cores. A blinded pathologist assessed each core and allocated a score based on the presence of: (A) no staining (score = 0), (B) weak membranous and/or cytoplasmic staining (score = 1) or (C) strong membranous and/or cytoplasmic staining (score = 2).

Cores were scored for FXYD3 immunolabelling intensity by a surgical pathologist (JT) who was blinded to all clinical outcomes. Patients were each given a score from 0 to 2 (Figure 1). A score of 0 was given where no staining was observed. A score of 1 was given where weak cytoplasmic and/or membranous staining was seen. A score of 2 was given where there was observed to be strong membranous and/or cytoplasmic staining.

### 2.4 Statistical Analysis

Categorical variables were compared using Fisher exact test. Survival curves were generated using Kaplan–Meier method. Comparative survival analyses were performed using log‐rank method. All statistical analyses were performed using GraphPad Prism 9. *p*‐values of < 0.05 were considered statistically significant.

## 3 Results

### 3.1 Baseline Characteristics

One hundred and eighty patients were included in the present analysis. The demographic and clinicopathological features are in Table 1. The rate of positive immunohistochemical FXYD3 expression was 80.5% (145/180 patients). Representative examples of FXYD3 immunoreactivity scoring are shown in Figure 1.

**TABLE 1:** Demographic and clinicopathological features of patients with primarily resected pancreatic ductal adenocarcinoma included in study cohort.

Patient Variables		Number of patients (*n*)
Gender	Female	97
	Male	83
Median age, years (range)	69 (34–87)	
T stage > 2	T1/2	117
	T3/4	63
Lymph node status	Positive	113
	Negative	67
Lymphovascular invasion	Positive	93
	Negative	63
	Unknown	24
Perineural invasion	Positive	123
	Negative	46
	Unknown	11
Grade > 2	Low (G1/2)	125
	High (G3/4)	55
FXYD3 expression	Negative	35
	Weak	68
	Strong	77

### 3.2 FXYD3 Expression and Overall Survival

**FIGURE 2:**
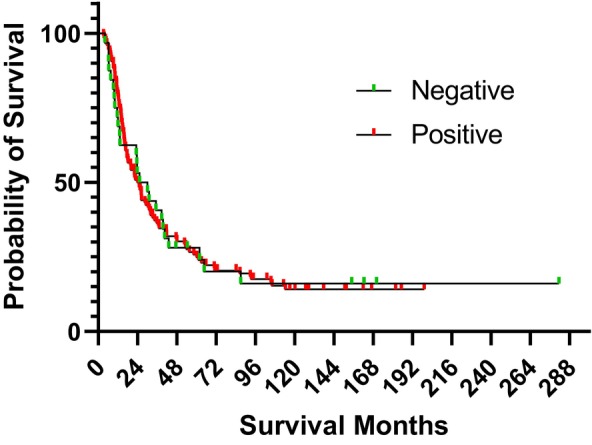
Survival analysis based on the FXYD3 expression. Kaplan–Meier curves for cumulative overall survival in patients who underwent primary resection of pancreatic ductal adenocarcinoma, with or without FXYD3 expression. There was no significant survival difference between patients with positive (red line; mOS 25 months; *n* = 128) and negative (green line; mOS 27.6 months; *n* = 32) immunohistochemical FXYD3 expression. Log‐rank *p*‐value = 0.9718.

Patients with less than 1 year follow up or who had mortality within 3 months of surgery were excluded from survival analysis. A total of 160 patients were included in survival analysis. There was no significant association between FXYD3 expression and duration of overall survival (FXYD3 negative 27.6 months vs. FXYD3 positive 25.00 months, log‐rank *p* = 0.9718; Figure 2).

### 3.3 FXYD3 and Histopathological Parameters

The association between FXYD3 positivity and histopathological parameters is demonstrated in Table 2. FXYD3 expression significantly correlated positively with late‐stage disease (OR 3.041, 95% CI 1.190–7.455, *p* = 0.0175). There was no significant association between FXYD3 staining and other clinicopathological parameters (T stage, lymph node status, perineural invasion, lymphovascular invasion and histological grade). As patients with Overall Stage III and IV as well as T Stage 3 and 4 could have potentially different prognostic outcomes, further analysis was performed to determine their individual association with FXYD3 positivity. Similar results were obtained, with overall stage being associated with FXYD3 positivity (*p* < 0.05) while there is no significant association with T stage (Table S1).

**TABLE 2:** Relationship between immunohistochemical FXYD3 expression and clinicopathological markers of PDAC tumour stage and grade.

Pathological variable	FXYD3 negative	FXYD3 positive	Odds ratio (95% CI)	*p*
Overall stage				
Early (I–II)	29	89	3.041 (1.190–7.455)	0.0175
Late (III–IV)	6	56		
Lymph node status				
Positive	18	95	0.5921 (002797–1.284)	0.2356
Negative	16	50		
T stage				
Early (T1/2)	23	94	1.040 (0.4940–2.296)	> 0.9999
Late (T3/4)	12	51		
Perineural invasion				
Yes	27	96	1.875 (0.7606–4.729)	0.2754
No	6	40		
Lymphovascular invasion				
Yes	18	95	0.5573 (0.2671–1.175)	0.1719
No	17	50		
Histological grade				
Low (G1/2)	22	94	0.5079 (0.2403–1.100)	0.0784
High (G3/4)	12	51		

*Note:* Categorical variables were compared using a Fischer exact test.

## 4 Discussion

The present study has demonstrated a high rate of immunohistochemical FXYD3 expression (80.5%) in PDAC. This study has also shown a lack of overall prognostic significance of FXYD3.

To our knowledge, this study is the largest to date examining FXYD3 expression and its prognostic significance in pancreatic cancer, but is limited by its retrospective nature and single centre cohort.

In recent years, ion channels and their modulators have become increasingly central to theories on the pathogenesis of neoplasia, influencing angiogenesis, accelerated cell cycle and impeded cell death [20]. FXYD3 was first identified as an overexpressed protein in murine breast tumours initiated by oncogenic *ras* and *neu* mutations, and was found to induce chloride conductance in *Xenopus* oocytes [6]. Subsequently, FXYD3 was characterised as a regulator of the Na/K‐ATPase [7], in part by protecting its beta 1 subunit from oxidative stress‐induced glutathionylation and pump inhibition [21].

Given the relationship between FXYD3 and the Na/K‐ATPase, there are numerous plausible mechanisms to explain the potential role of FXYD3 in cancer progression. For example, FXYD3 could influence the induction of growth‐related genes by Na/K‐ATPase [22], TGF‐beta 1‐induced epithelial‐mesenchymal transition [18, 23] or cellular protection against pro‐apoptotic pathways such as those induced by cardiac glycosides via the Na/K‐ATPase [24, 25]. However, the extent to which FXYD3 contributes to these pathways has not yet been investigated.

The overexpression of FXYD3 in PDAC has been consistently demonstrated through several studies of gene and protein expression [15, 16, 17, 18]. However, the literature to date demonstrates that FXYD3 may have mixed roles in the progression and inhibition of PDAC. One study demonstrates that FXYD3 silencing does significantly slow down the proliferation of T3M4 PDAC cell lines [18], leading to the conclusion that FXYD3 expression potentiates tumour growth. In contrast, large‐scale transcriptomic studies evaluating PDAC have shown that FXYD3 overexpression is linked to more favourable pancreatic cancer clusters such as the Collisson ‘classical’ PDAC subtype [26] and the Bailey non‐‘squamous’ PDAC subtypes (aberrantly differentiated endocrine exocrine, pancreatic progenitor and immunogenic) [27].

The present study has demonstrated immunohistochemical FXYD3 expression is not a prognostic biomarker in chemo‐naive resected PDAC. However, given that siRNA‐induced suppression of FXYD3 in breast cancer cells that overexpress FXYD3 amplifies effects of doxorubicin or γ‐radiation on cell death and apoptosis, overexpression of FXYD3 is a potentially useful treatment target [28]. With the high prevalence of FXYD3 expression in PDAC shown here, there might also be significant scope for FXYD3 as a treatment target as an adjunct to systemic pancreatic cancer therapies. Of the 3 β‐subunits of the α/β Na/K‐ATPase heterodimer only β1 is susceptible to glutathionylation and mutation of a specific Cysteine eliminates its protection against β1 subunit glutathionylation. Glutathionylation disrupts α1‐β1 binding and causes Na/K‐ATPase inhibition [29]. However, the β1 subunits' extracellular domains form between‐cell‐bonds while the α1 subunit is anchored to the cytoskeleton. This effectively makes the α1/β1 heterodimer a cell adhesion complex [30]. Targeting cell adhesion is potentially useful in cancer [31] and exposing BxPC‐3 pancreatic cancer cells that overexpress FXYD3 to a Cysteine‐mutated peptide derivative of full‐length FXYD3 displaces the wild‐type protein, increases doxorubicin‐induced β1 subunit glutathionylation and greatly enhances doxorubicin cytotoxicity. Exposure to a peptide that is not Cysteine‐mutated adds to the protective effect of the native FXYD3 protein. In contrast, exposing PANC‐1 pancreatic cancer cells that do not express FXYD3 to either peptide has no effect on doxorubicin cytotoxicity [32]. A peptide‐drug derived from the Cys‐mutated FXYD3 peptide potentially might sensitise highly prevalent pancreatic cancers overexpressing FXYD3 to systemic therapies. Moreover, since FXYD3 is detectable in plasma [33], it might be useful to explore the role of FXYD3 expression as a blood‐based biomarker in predicting response to neoadjuvant chemo‐ and/or chemoradiotherapy in future studies.

## 5 Conclusions

Immunohistochemical FXYD3 expression is not significantly associated with overall survival in chemo‐naive resected PDAC. However, FXYD3 is expressed in the vast majority of PDAC tumours, and therefore its utility as a therapeutic target should continue to be explored.

## Author Contributions

**Nathalie B. Rasko:** formal analysis (equal), investigation (equal), writing – original draft (equal), writing – review and editing (equal). **Christopher B. Nahm:** formal analysis (equal), writing – review and editing (equal). **John Turchini:** formal analysis (equal), writing – review and editing (equal). **Rachel Teh:** formal analysis (equal), writing – review and editing (equal). **Helge Rasmussen:** conceptualization (equal), writing – review and editing (equal). **Sooin Byeon:** data curation (equal), writing – review and editing (equal). **Sumit Sahni:** formal analysis (equal), writing – review and editing (equal). **Jaswinder S. Samra:** supervision (equal), writing – review and editing (equal). **Anthony J. Gill:** resources (equal), supervision (equal), writing – review and editing (equal). **Anubhav Mittal:** resources (equal), supervision (equal), writing – review and editing (equal).

## Ethics Statement

Ethical approval was obtained from the Northern Sydney Local Health District Human Research and Ethics Committee (Master Protocol # 2019/ETH08639 and associated LNR# 2023ETH01069). A waiver of consent was obtained from NSLHD HREC to use archived tissue blocks under NSW Human Tissue Act 1983.

## Conflicts of Interest

The authors declare no conflicts of interest.

## Supporting information


**Table S1:** Relationship between immunohistochemical FXYD3 expression and Overall Stage and Tumour Stage. Categorical variables were compared using a Fischer exact test.

## Data Availability

Data will be available on reasonable request from corresponding author.
