# Supplementary material for: FXYD3 Is Frequently Expressed in Pancreatic Ductal Adenocarcinoma but Does Not Predict Survival
Source: Cancer Med. 2025 Jan 9;14(1):e70500. doi: 10.1002/cam4.70500 (PMC11714220; doi:10.1002/cam4.70500)
Supplement: Supplementary file 1 — Table S1: Relationship between immunohistochemical FXYD3 expression and Overall Stage and Tumour Stage. Categorical variables were compared using a Fischer exact test. [file CAM4-14-e70500-s001.docx]

**Supplementary Table 1.** Relationship between immunohistochemical FXYD3 expression and Overall Stage and Tumor Stage. Categorical variables were compared using a Fischer exact test.

| **Pathological Variable** | **FXYD3 negative** | **FXYD3**  **positive** | **P-value** |
| --- | --- | --- | --- |
| **Overall Stage** | | | |
| I-II | 29 | 89 | 0.0415 |
| III | 6 | 46 |  |
| IV | 0 | 10 |  |
| **T Stage** | | | |
| T1/2 | 23 | 94 | 0.68 |
| T3 | 12 | 45 |  |
| T4 | 0 | 6 |  |
